# Longitudinal follow-up of autophagy and inflammation in brain of APPswePS1dE9 transgenic mice

**DOI:** 10.1186/s12974-014-0139-x

**Published:** 2014-08-27

**Authors:** Arnaud François, Agnès Rioux Bilan, Nathalie Quellard, Béatrice Fernandez, Thierry Janet, Damien Chassaing, Marc Paccalin, Faraj Terro, Guylène Page

**Affiliations:** EA3808 molecular Targets and Therapeutic of Alzheimer’s disease, University of Poitiers, 1 Rue Georges Bonnet, TSA 51106 86073 Poitiers, Cedex 9 France; Pathology Department, Poitiers University Hospital, Poitiers, F-86021 France; Geriatrics Department, Poitiers University Hospital, Poitiers, F-86021 France; CMRR, Poitiers University Hospital, Poitiers, F-86021 France; CIC-P 1402, Poitiers University Hospital, Poitiers, F-86021 France; Laboratory of Histology and Biology, Faculty of Medicine, University of Limoges, Limoges, F-87025 France; Service d’histologie et de cytogénétique, Hôpital de la Mère et de l’Enfant, Limoges, F-87025 France

**Keywords:** Alzheimer, Beclin-1, IL-1β, TNF-α, Transgenic mouse model

## Abstract

**Background:**

In recent years, studies have sought to understand the mechanisms involved in the alteration of autophagic flux in Alzheimer's disease (AD). Alongside the recent description of the impairment of lysosomal acidification, we wanted to study the relationships between inflammation and autophagy, two physiological components deregulated in AD. Therefore, a longitudinal study was performed in APPswePS1dE9 transgenic mice at three, six and twelve months of age.

**Methods:**

Autophagic markers (Beclin-1, p62 and LC3) and the activation of mammalian Target of Rapamycin (mTOR) signaling pathway were quantified by western blot. Cytokine levels (IL-1β, TNF-α and IL-6) were measured by ELISA. Transmission electron microscopy was performed to detect autophagic vacuoles. Mann-Whitney tests were used to compare wild-type (WT) versus APPswePS1dE9 mice. Longitudinal changes in parameters were analyzed with a Kruskal-Wallis test followed by a *post-hoc* Dunn’s test. Correlation between two parameters was assessed using a Spearman test.

**Results:**

Compared to 12-month old WT mice, 12-month old APPswePS1dE9 mice had higher levels of IL-1β and TNF-α, a greater inhibition of the mTOR signaling pathway and lower levels of Beclin-1 expression both in cortex and hippocampus. Regarding the relationship of the various parameters in 12-month old APPswePS1dE9 mice, Beclin-1 rates were positively correlated with IL-1β and TNF-α levels. And, on the contrary, TNF-α levels were inversely correlated with the levels of mTOR activation. Altogether, these results suggest that inflammation could induce autophagy in APPswePS1dE9 mice. However, these transgenic mice displayed a large accumulation of autophagic vesicles within dystrophic neurons in cortex and hippocampus, indicating a terminal failure in the autophagic process.

**Conclusions:**

This first demonstration of relationships between inflammation and autophagy in *in vivo* models of AD should be taken into account in new therapeutic strategies to prevent inflammation and/or stimulate autophagy in advanced neurodegenerative process such as AD.

## Introduction

Alzheimer's disease (AD) is a progressive dementia with atrophy, senile plaques of fibrillogenic beta-amyloid (Aβ) and intraneuronal neurofibrillary tangles with hyperphosphorylated tau. In 1988, it was discovered that microglia around Aβ deposits expressed major histocompatibility complex type II, a marker for activated immune cells [1-4]. However, the notion that the CNS is an immunologically privileged organ is questioned and microglia are generally recognized as the brain’s resident players in innate immune/inflammatory responses in AD. Like microglia, reactive astrocytes encircle Aβ deposits in a manner reminiscent of glial scarring [5-7]. In addition, many molecular mediators are involved in the inflammatory response in AD, including complement factors, cytokines, chemokines, pattern recognition receptors, scavenger receptors, cyclooxygenases and reactive oxygen species [8-11]. Several data from literature are in favor of a deleterious role of inflammation, in particular the activation of the inflammasome [10,12-15], but more research is needed to better understand its role according to the stage of the disease and the many failures of anti-inflammatory therapies in patients with AD.

It is known that peripheral inflammation plays a role in cell proteolysis; in particular, molecular relationships were identified with macroautophagy (called autophagy) which degrades long-lived proteins and organelles [16-21]. Autophagy has an anti-inflammatory function as demonstrated in a mouse model of Crohn’s disease [22] and many studies have shown that autophagy targets ubiquitinated aggregated inflammasome components for destruction [23-26]. Autophagy may have a protective role against the development of a number of neurodegenerative diseases [27,28]. Autophagy deregulation was initially linked to AD when electron microscopy studies revealed the accumulation of autophagic vacuoles in the brains of AD patients [29,30]. Compelling evidence indicates that Aβ is generated in autophagic vacuoles during autophagy, suggesting that autophagy may exacerbate AD pathogenesis by increasing Aβ levels [31-33]. Furthermore, clearance of autophagic vacuoles is impaired in AD brains [34] and one probable contributor to autophagy deficiency in AD appears to be Beclin-1, whose expression is strongly reduced in the brains of AD patients to levels that would be predicted to impair autophagosome synthesis [35,36]. The relationships between autophagy and inflammation in AD have not yet been explored *in vivo*. This present study aims to track key inflammatory cytokine actors (IL-1β, TNF-α and IL-6) and autophagy markers (Beclin-1, p62 and microtubule-associated protein 1/light chain 3 (LC3)) in APPswePS1dE9 transgenic mice. These mice are used extensively in research and have a significant inflammatory response at 12 months. However, their autophagic status has never been followed over time.

## Materials and methods

### Chemical products

Sodium fluoride (NaF), phenylmethylsulfonyl fluoride (PMSF), protease and phosphatase inhibitor cocktails, dithiothreitol (DTT), paraformaldehyde (PFA) and all reagent-grade chemicals for buffers were purchased from Sigma (St Quentin Fallavier, France); sodium pentobarbital from CEVA, Animal Health (Libourne, France); NuPAGE® LDS 4X Sample Buffer, NuPAGE® Sample Reducing Agent (10X), Novex® 4 to 20% Tris-Glycine Mini gels, NuPAGE® 3 to 8% Tris-Acetate gels, Novex® Tris-Glycine SDS Running Buffer and NuPAGE® Tris-Acetate SDS Running Buffer, NuPAGE® Antioxidant, Seeblue® Plus2 pre-stained standard, iBlot® Gel Transfer Device (EU), Quant-it® protein assay from Gibco-Invitrogen (Fisher Bioblock Scientific distributor, Illkirch, France); 4X Laemmli Sample Buffer, 4 to 15% mini-PROTEAN® TGX™ gels, Tris-Glycine Running Buffer and Trans-Blot® Turbo™ Transfer System from Bio-Rad (Marnes-la-Coquette, France).

For western blot, primary antibodies and secondary anti-rabbit IgG antibody conjugated with horseradish peroxidase (HRP) were purchased from Cell Signalling (Ozyme, Saint-Quentin-en-Yvelines, France) except sequestosome 1 (p62/SQMT1) from MBL (CliniSciences distributor, Nanterre, France), anti-β tubulin from Sigma (St Quentin Fallavier, France), HRP-conjugated anti-mouse IgG from Amersham Biosciences (Orsay, France), IgG- and protease-free BSA from Jackson ImmunoResearch Europe Ltd (Interchim distributor, Montluçon, France). Molecular biology reagents were obtained from Promega (Charbonnières, France).

### Animals

Male hemizygote B6C3-Tg (APPswe, PS1dE9)85Dbo (stock #004462) and female wild-type (WT) mice (B6C3F1, stock #10010) were obtained from Jackson Laboratories (Bar Harbor, ME USA) and bred to create colonies of APPswePS1dE9 and WT mice. As described in the website of Jackson Laboratories, two expression plasmids (Mo/HuAPP695swe and PS1-dE9) were designed to each be controlled by independent mouse prion protein (PrP) promoter elements, directing transgene expression predominantly to central nervous system (CNS) neurons. The Mo/HuAPP695swe transgene expresses a ‘humanized’ mouse amyloid beta (A4) precursor protein gene modified at three amino acids to reflect the human residues and further modified to contain the K595N/M596L mutations linked to familial Alzheimer’s disease (FAD). The PS1dE9 transgene expresses a mutant human presenilin 1 carrying the exon-9-deleted variant (PSEN1dE9) associated with FAD. Occasional deposits can be found in mice as young as six months of age and plaques are abundant in hippocampus and cortex by nine months of age. Furthermore, in this APPswePS1dE9 mouse model of AD, amyloid plaques and its associated inflammatory response develop at early stage of the life and progressively increase with age [36,37].

Mice derived from crosses of 12 breeders. At weaning, all mice were tattooed (ISO Tiny chip, Biolog-id, Bernay, France), genotyped by PCR analysis of tail biopsies according to the manufacturer’s recommended protocol (KAPA Mouse Genotyping HotStart Kit, CliniSciences, Nanterre, France).

The use of animals for this study was approved by the Ethical and Animal Care Committee at ‘La direction départementale de la protection de la population (DDPP)’ (registration number: 06.12). An agreement was obtained from The High Council of Biotechnology for transgenic animals in 2010 (5418 number agreement). All animal care and experimental procedures conformed with the French Décret number 2013-118, 1 February 2013 NOR: AGRG1231951D in accordance to European Community guidelines (directive 2010/63/UE for the Care and Use of Laboratory Animals). All efforts were made to minimize animal suffering as well as the number of animals used. The animals were housed in a conventional state under adequate temperature (23 ± 3°C) and relative humidity (55 ± 5%) control with a 12/12 hour reversed light/dark cycle, and provided with food and water *ad libitum*.

### Brain tissue preparation for biochemical analysis

At 3, 6 and 12 months of age, mice were transcardially perfused with PBS (154 mM NaCl, 1.54 mM KH_2_PO_4_, 2.7 mM Na_2_HPO_4_.7H_2_O, pH 7.2) after deep anesthesia with pentobarbital (80 mg/kg, intraperitoneally (ip)). Brains were rapidly removed and dissected on ice. Cortex and hippocampus were homogenized using 10 up-and-down strokes of a prechilled Teflon-glass homogenizer in 20 volumes of lysis buffer (25 mM Tris-HCl, 150 mM NaCl, 1 mM EDTA, pH 7.4) and supplemented with 50 mM NaF, 1 mM PMSF, protease and phosphatase inhibitor cocktails (50 μL/g of tissue and 10 μL/mL of lysis buffer, respectively). Lysates were sonicated and centrifuged at 15,000 *g* for 15 minutes at 4°C. The resulting supernatants were collected for Quant-it® protein assay according to the manufacturer’s protocol. Samples were stored at −20°C until ELISA and immunoblotting described below. For q-PCR, a piece (10 mg of tissue) of cortex and hippocampus was immediately homogenized in RNA lysis buffer available in the kit from Promega by using sterile 25-gauge needle and syringe. These samples were stored at −80°C until RNA extraction.

### TaqMan real-time PCR

Total RNA was isolated from cortex and hippocampus by using SV Total RNA Isolation System according to the manufacturer’s instructions (Promega, Charbonnieres, France). Concentration of total RNA was measured by using a nanodrop (Nanodrop 2000 spectrophotometer, ThermoScientific (Villebon sur Yvette, France). Total RNA isolate (500 ng) was converted into first strand cDNA using ImProm-II™ Reverse Transcription System (Promega, Charbonnieres, France) .

The TaqMan probes and primers were designed according to the sequence of murine p62, LC3, Beclin-1 and S6 ribosomal protein genes using the Primer Express software version 2.0 (ABI Applied Biosystems, Foster City, CA, USA). Primers p62-F (5′-CTGCACAGGGAACACAGCAA-3′) and p62-R (5′ GCCAGCGGCTATGAGAGAAG-3′), LC3-F (5′-TCGCCGACCGCTGTAAG-3′) and LC3-R (5′-CTCGATGATCACCGGGATCT-3′), Beclin-F (5′-CTGCACAGGGAACACAGCAA-3′) and Beclin-R (5′-GCCAGCGGCTATGAGAGAAG-3′) and S6-F (5′-AAGTCGGGCCTCTTTTTCGT-3′) and S6-R (5′ GGGAAGGAGATGTTCAGCTTCA-3′) were designed to yield respectively a 79 bp, 71 bp, 146 bp and 73 bp fragments of the *p62*, *LC3*, *Beclin-1* and *S6* ribosomal protein genes. The TaqMan probes p62-P (5′-TCCCAACCCCTTTGGCCACCTCT-3′), LC3-P (5′-TCCGCGACCAGCACCCCAG-3′), Beclin-P (5′- CCTTCCACATCTGGCACAGCGGA-3′); S6-P (5′-CCTCCCAGGCGCTCGGCTG-3′); contained 6-carboxy-fluorescein (FAM) reporter dye at the 5′ end and 6-carboxytetramethylrhodamine (TAMRA) fluorescent quencher at the 3′ end. Primers and probes were commercially synthesized by Eurogentec (Angers, France). TaqMan real-time PCR was performed in an ABI 7500 real-time PCR system (Applied Biosystems, Foster City, CA, USA). Amplification reaction contained 2X TaqMan Universal Master Mix (Applied, Villebon-sur-Yvette, France), 900 nM of each primer (p62-F and p62-R or LC3-F and LC3-R or Beclin-1-F and Beclin-R), 900 nM of probe and cDNA template and nuclease-free water to a final volume of 13 μL. The thermal profile consisted of 50°C for 2 minutes, followed by 95°C for 10 minutes and 40 cycles of 95°C for 15 seconds and 59°C for 1 minute. Fluorescence was measured once *per* cycle at the end of the 59°C segment.

The standard curve was generated using 10-fold serial dilutions with ddH_2_O from 10^−1^ to 10^−6^ cDNA synthesized from RNA of lipopolysaccharide (LPS)-treated mouse spleen (positive control). The PCR conditions for this standard curve were adopted to perform the further reactions to estimate transcriptional expression of p62, LC3 and Beclin-1 by TaqMan real-time PCR assay. For each gene and structure, results were normalized relatively to mean of WT mice.

### ELISA

Commercially available ELISA kits were used for assessing IL-1β (sensitivity: 16 pg/mL) TNF-α (sensitivity: 4 pg/mL) and IL-6 (sensitivity: 2 pg/mL) according to the manufacturers' instructions (BioLegend, Ozyme, Saint-Quentin-en-Yvelines, France). The range of analysis was between 31.3 to 2,000 pg/mL for IL-1β and 7.8 to 500 pg/mL for TNF-α and IL-6. Homogenates from brain tissue (50 mg of tissue/mL) were added in each well of pre-coated plates and all steps were performed at room temperature (RT). The enzymatic reaction was stopped after 15 minutes incubation with tetramethylbenzidine (TMB) substrate by adding 2 N H_2_SO_4_ and the optical density (OD) was read at 450 nm within 30 minutes, using the Multiskan® spectrum spectrophotometer (ThermoScientific, Villebon sur Yvette, France). The cytokine levels were then calculated by plotting the OD of each sample against the standard curve. The intra- and inter-assay reproducibility was > 90%. OD values obtained for duplicates that differed from the mean by greater than 10% were not considered for further analysis. For convenience, all results are expressed in pg/mg protein.

### Immunoblottings

Samples (40 μg proteins) were prepared for electrophoresis by adding NuPAGE® 4X LDS Sample Buffer and NuPAGE® Sample Reducing Agent (10X). Samples were then heated up 100°C for 5 minutes, loaded into Novex® 4 to 20% Tris-Glycine mini Gels, run at 150 V for 60 minutes in Novex® Tris-Glycine SDS Running Buffer and in NuPAGE® 3 to 8% Tris-Acetate Gels, run at 125 V for 120 minutes in NuPAGE® Tris-Acetate SDS Running Buffer containing NuPAGE antioxidant. Gels were transferred to nitrocellulose membranes using the iBlot® Dry blotting system set to program 20 V for 7 minutes. For LC3 analysis, we used Trans-Blot® Turbo™ Transfer System (25 V, 3 minutes for 0.2 μm nitrocellulose MISI format) after protein gel electrophoresis of samples prepared in 4X Laemmli Sample Buffer and loaded into 4 to 15% mini-PROTEAN® TGX™ gels with Tris-Glycine SDS Running Buffer.

Membranes were washed for 10 minutes in Tris-buffered saline/Tween (TBST: 20 mM Tris-HCl, 150 mM NaCl, pH 7.5, 0.05% Tween 20) and not specific antigenic sites were blocked for 2 hours in TBST containing 5% BSA.

Blots were incubated with primary antibody in blocking buffer overnight at 4°C. Antibodies used were rabbit anti-P_S2448_-mTOR, anti-total mTOR, anti-P_T389_-p70S6K, anti-total p70S6K, anti-Beclin-1, anti-p62, anti-LC3, all at 1:500 dilution factor. Membranes were washed twice with TBST and then incubated with the HRP-conjugated secondary antibody anti-rabbit IgG (1:1,000), during 1 hour at RT. Membranes were washed again and exposed to the chemiluminescence Luminata Forte Western HRP Substrate (Millipore, Saint-Quentin-en-Yvelines, France) followed by signal capture with the Gbox system (GeneSnap software, Syngene, Ozyme distributor, Saint-Quentin-en-Yvelines, France). After 2 washes in TBST, membranes were probed with mouse antibody against β-tubulin (1:10,000) overnight at 4°C. They were then washed with TBST, incubated with HRP-conjugated secondary antibody anti-mouse (1:1,000) for 1 hour, exposed to the chemiluminescence Luminata classico substrate (Millipore, Saint-Quentin-en-Yvelines, France) and signals were captured. Automatic image analysis software is supplied with Gene Tools (Syngene, Ozyme distributor, Saint-Quentin-en-Yvelines, France). Protein/β-tubulin *ratios* were calculated and shown in the corresponding figures. Phospho-protein/total protein *ratios* were calculated to evaluate rates of protein activation.

### Transmission electron microscopy (TEM)

For brain TEM, four mice were transcardially perfused with PBS followed by 4% PFA perfusion after deep anesthesia with pentobarbital (80 mg/kg, ip). Brains were rapidly removed on ice and thin sagittal sections were isolated and fixed with 3% glutaraldehyde in PBS (0.1 M; pH = 7.4) for 2 hours at 4°C. Samples (2 mm^3^ of tissue in cortex and hippocampus) were then washed three times (3 × 10 minutes) in PBS before being post-fixed in 1% osmium tetroxide in PBS for 1 hour at 4°C, processed through a graded acetone series, embedded in Araldite (Fluka, Buchs, Switzerland) and polymerized overnight at 60°C. Thin sections (60 nm) were cut with a diamond knife on Reichert Ultracut (Leica, Nanterre, France) S, recovered on Cu grids and contrasted with uranyl acetate (4%) and lead citrate and were observed under a JEOL 1010 transmission electron microscope (Jeol Ltd, Tokyo, Japan).

### Statistical analysis

For biochemical analysis, results are expressed as means ± SEM. To compare quantitative variables between WT mice and APPswePS1dE9 mice, Mann-Whitney tests were used. Comparisons between the three classes of age in both groups of mice were performed using a Kruskal-Wallis test followed by a *post-hoc* Dunn’s test. Correlations between two quantitative parameters in APPswePS1dE9 mice were estimated by Spearman tests (GraphPad Instat, GraphPad Software, San Diego, CA, USA). The level of significance was *P* < 0.05.

## Results

### Longitudinal monitoring of cytokines in the brain of APPswePS1dE9 mice

**Figure 1:**
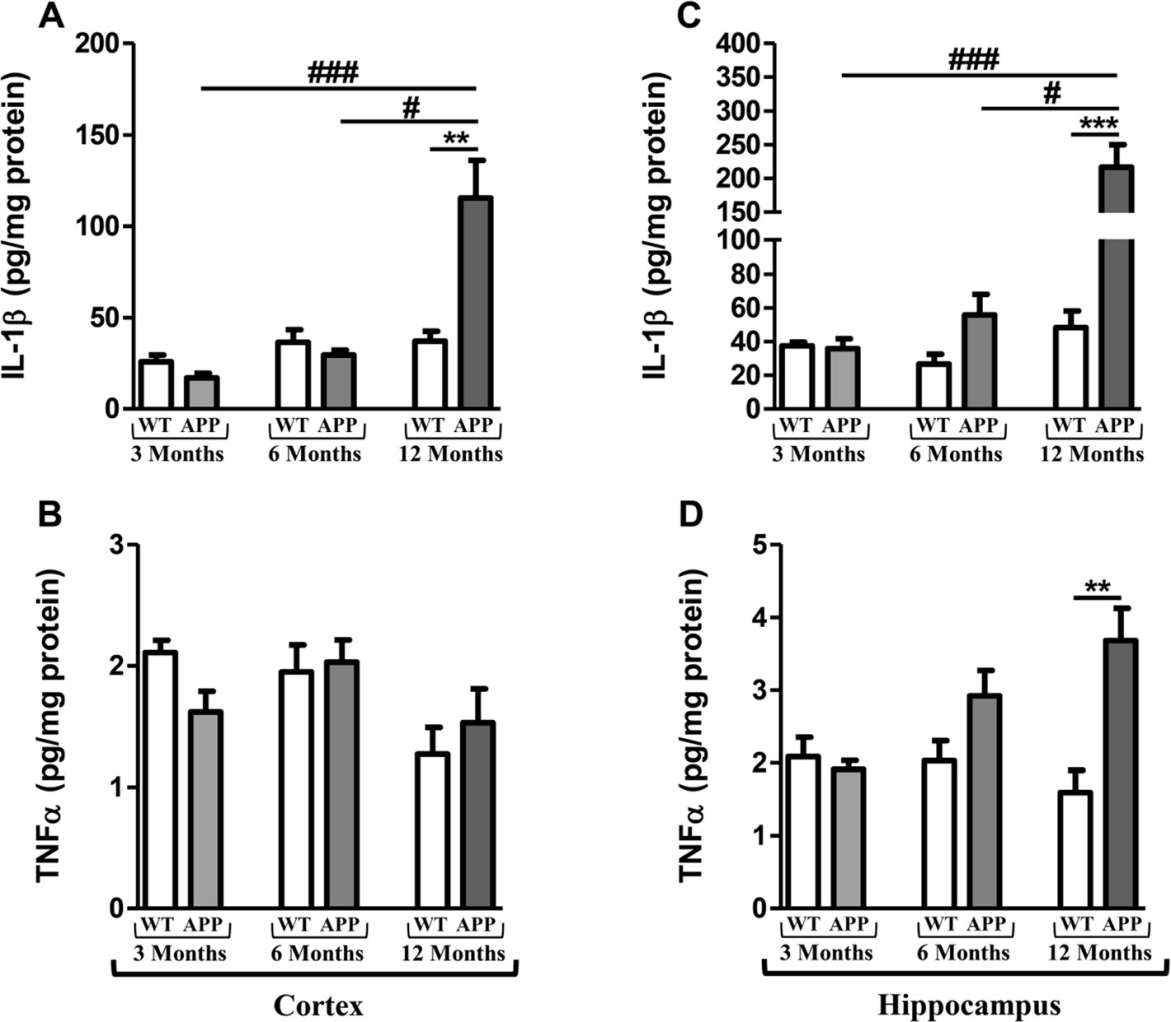
**Longitudinal monitoring of cytokines in the brain of APPswePS1dE9 mice.** IL1-β and TNF-α levels were measured by ELISA in cortex **(A and B)** and hippocampus **(C and D)** of APPswePS1dE9 (APP) and wild-type mice (WT) at three, six and twelve months of age. Cytokine levels were expressed in pg/mg protein. Results are mean ± SEM of 12 mice in each group. Comparison between APP and WT mice at each age by a Mann-Whitney test: ^**^ *P* < 0.01, ^***^ *P* < 0.001; comparison of the 3 classes of age (Kruskall-Wallis test) followed by two-by-two comparisons (Dunn’s multiple comparison test in APP mice): ^#^ *P* < 0.05 for 6-month old versus 12-month old APP mice, ^###^ *P* < 0.001 for 3-month old versus 12-month old APP mice.

IL-1β levels in APPswePS1dE9 mice were significantly higher in cortex (3.1-fold) and hippocampus (4.5-fold) than those of WT mice only at 12 months of age. Furthermore, 12-month old APPswePS1dE9 mice produced more IL-1β than 3-month old APPswePS1dE9 mice (6.8-fold in cortex, 6-fold in hippocampus) and 6-month old APPswePS1dE9 mice (3.25-fold in cortex and 3.9-fold in hippocampus), (Figure 1A and C). TNF-α levels were higher in 12-month old APPswePS1dE9 mice than in respective WT mice (2.3-fold) in hippocampus only (Figure 1B and D). For IL-6 levels, no difference between the two groups of mice at 12 months of age was observed in both brain areas (cortex: 2.13 ± 0.33 and 2.94 ± 0.59 pg/mg protein for APPswePS1dE9 and WT mice, respectively; hippocampus: 3.30 ± 0.37 and 2.82 ± 0.49 pg/mg protein for APPswePS1dE9 and WT mice, respectively).

### Changes in activation of the mTOR signaling pathway

mTOR activation leads to phosphorylation of various substrates, in particular p70S6K at T389, a ribosomal S6 kinase involved in ribogenesis [38,39]. Furthermore, it is also well known that autophagy is inhibited by the activation of the mTOR signaling [40].

**Figure 2:**
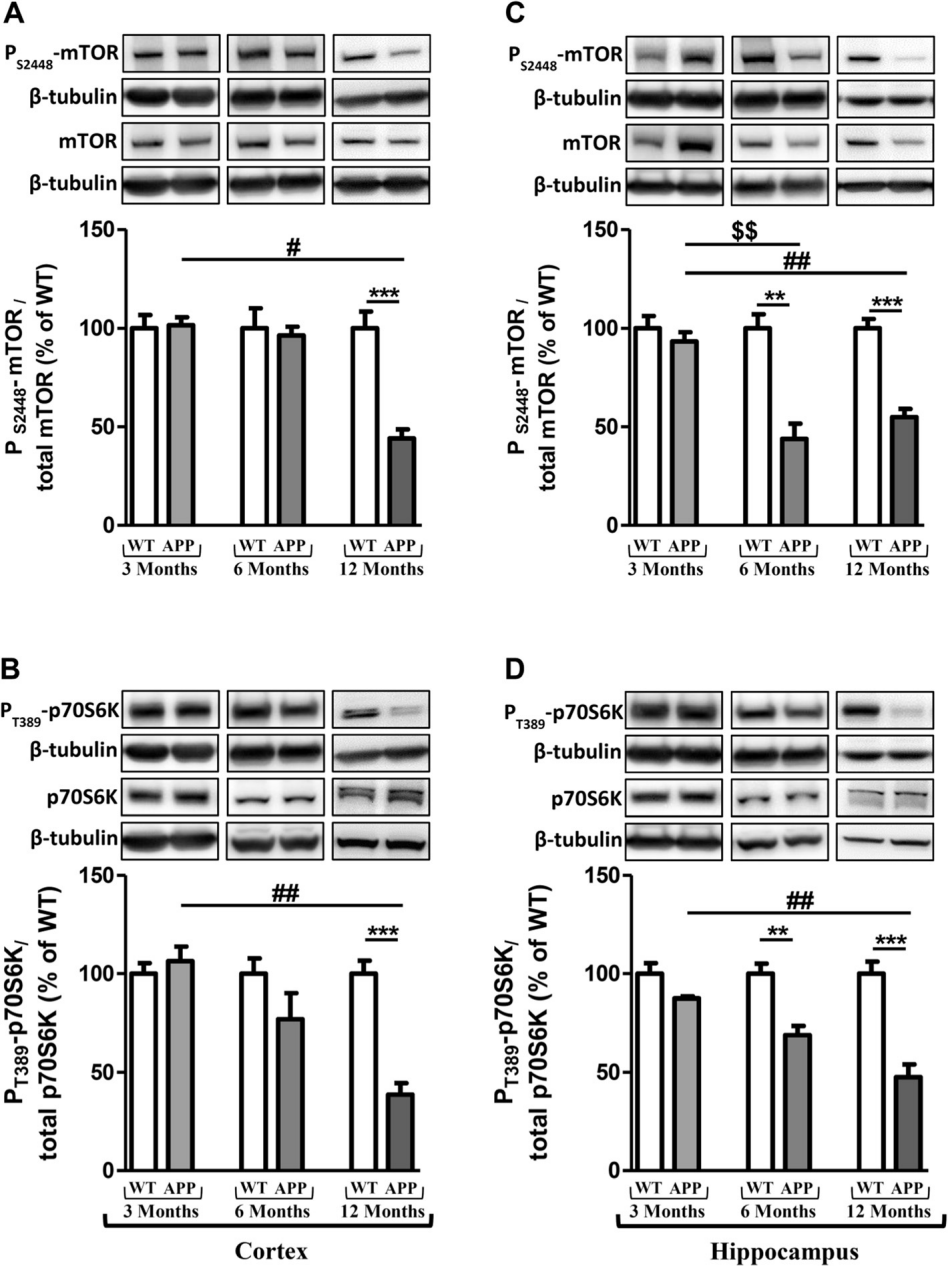
**Changes in activation of the mammalian Target of Rapamycin (mTOR) signaling pathway.** Representative immunoblots of P_S2448_-mTOR (289 kDa), total mTOR (289 kDa), P_T389_-p70S6K (70 kDa), total p70S6K (70 kDa) in cortex **(A and B)** and hippocampus **(C and D)** of wild-type (WT) mice and APPswePS1dE9 (APP) at 3, 6 and 12 months of age. Semi-quantitative analysis of immunoblot was performed using Gene Tools software (Syngene, Ozyme, Saint-Quentin-en-Yvelines, France). The immunoreactivity of protein was normalized to β-tubulin (55 kDa) immunoreactivity. The results are expressed as arbitrary units (% of WT). Results are mean ± SEM of 12 mice. Comparison between APP and WT mice at each age by a Mann-Whitney test: ^**^ *P* < 0.01, ^***^ *P* < 0.001; comparison of the 3 classes of age (Kruskall-Wallis test) followed by two-by-two comparisons (Dunn’s multiple comparison test in APP mice): ^$$^ *P* < 0.01 for 3-month old versus 6-month old APP mice, ^#^ *P* < 0.05, ^##^ *P* < 0.01 for 6-month old versus 12-month old APP mice.

In APPswePS1dE9 mice, the activation of mTOR and p70S6K decreased with age (Figure 2). Indeed, significant decreases were observed in 12-month old APPswePS1dE9 mice compared to WT mice (56% and 63.7% in cortex, 41.6% and 53.2% in hippocampus for mTOR and p70S6K, respectively). Furthermore, the activation of mTOR and p70S6K in twelve-month old APPswePS1dE9 mice was significantly different compared to transgenic mice at three and six months of age (Figure 2). Interestingly, the mTOR signaling pathway impairment was observed from 6-month old APPswePS1dE9 mice in hippocampus (decrease in 58.8% and 36% for mTOR and p70S6K compared to WT mice, respectively) and mTOR activation in 12-month old APPswePS1dE9 mice was significantly different compared to 3-month old APPswePS1dE9 mice (Figure 2C and D).

### Longitudinal monitoring of autophagy markers in the brain of APPswePS1dE9 mice

**Figure 3:**
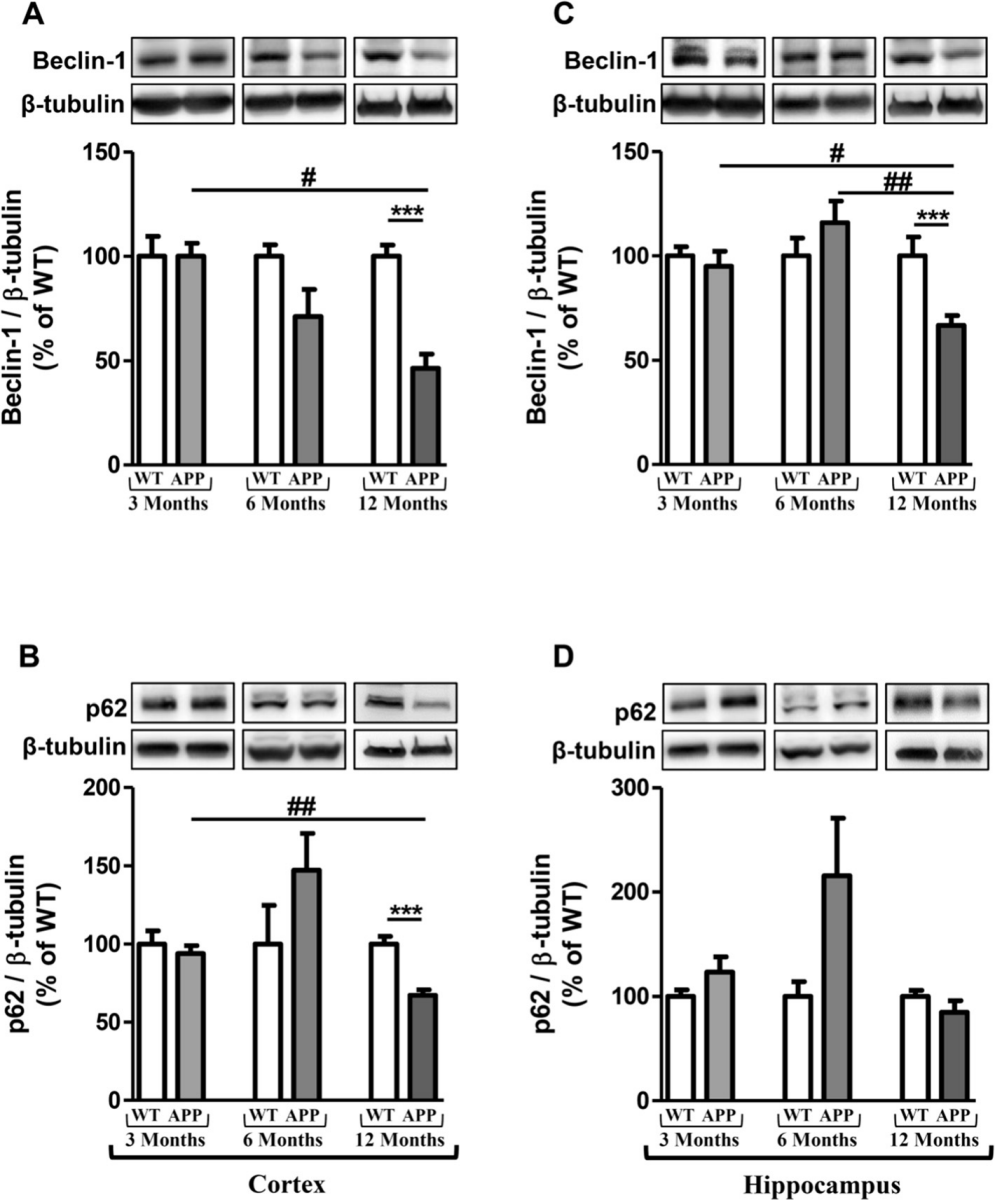
**Longitudinal monitoring of Beclin-1 and p62 in the brain of APPswePS1dE9 mice.** Representative immunoblots of Beclin-1 (60 kDa) and p62 (62 kDa) in cortex (**A** and **B**, respectively) and hippocampus (**C** and **D**, respectively) of wild-type (WT) and APPswePS1dE9 (APP) mice at 3, 6 and 12 months of age. Semi-quantitative analysis of immunoblot was performed using Gene Tools software (Syngene, Ozyme, Saint-Quentin-en-Yvelines, France). The immunoreactivity of protein was normalized to β-tubulin (55 kDa) immunoreactivity. The results are expressed as arbitrary units (% of WT). Results are mean ± SEM of 12 mice. Comparison between APP and WT mice at each age by a Mann-Whitney test: ^***^ *P* < 0.001; Comparison of the three classes of age (Kruskall-Wallis test) followed by two-by-two comparisons (Dunn’s multiple comparison test in APP mice): ^#^ *P* < 0.05, ^##^ *P* < 0.01 compared to 12-month old APPswePS1dE9.

To explore autophagy in both groups of mice, immunoblottings of Beclin-1, p62, LC3-I and LC3-II were performed. The levels of expression of Beclin-1, which is a key component in the initiation of autophagosome formation [41], decreased in 12-month old APPswePS1dE9 mice (57% and 36.8% in cortex and hippocampus compared to 12-month old WT mice, respectively). Regarding autophagy variations during the life in APPswePS1dE9, the levels of Beclin-1 expression in twelve-month old APPswePS1dE9 mice were also significantly lower than those of six-month old APPswePS1dE9 mice in hippocampus and three-month old APPswePS1dE9 mice in both brain areas (Figure 3A and C).

**Figure 4:**
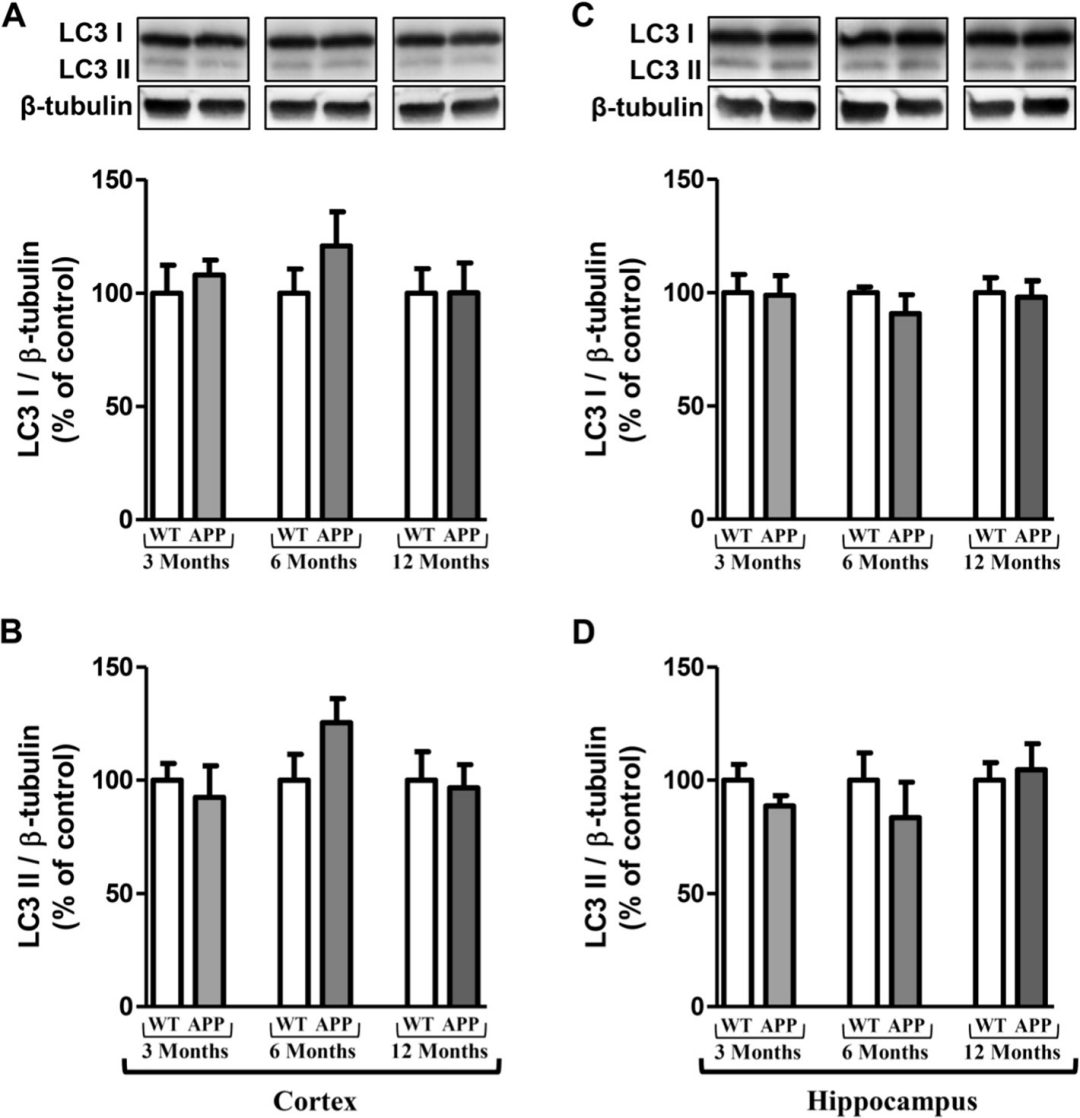
**Longitudinal monitoring of LC3 in the brain of APPswePS1dE9 mice.** Representative immunoblots of LC3-I (18 kDa), LC3-II (16 kDa) in cortex (**A** and **B**, respectively) and hippocampus (**C** and **D**, respectively) of wild-type (WT) and APPswePS1dE9 (APP) mice at 3, 6 and 12 months of age. Semi-quantitative analysis of immunoblot was performed using Gene Tools software (Syngene, Ozyme, Saint-Quentin-en-Yvelines, France). The immunoreactivity of protein was normalized to β-tubulin (55 kDa) immunoreactivity. The results are expressed as arbitrary units (% of WT). Results are mean ± SEM for 12 mice.

p62 is an autophagic receptor which recognizes ubiquitinylated proteins and interacts with LC3-II at the forming autophagosome [42]. LC3 is present in free cytoplasmic form as LC3-I which, when is associated to phosphatidylethanolamine (through an ubiquitin-like conjugation reaction) of the membrane of autophagosome, produces LC3-II form, a useful marker of autophagic membranes [42]. Results showed that the p62 expression decreased in cortex of APPswePS1dE9 mice at 12 months of age (30% compared to WT mice) and was significantly different compared to 3-month old APPswePS1dE9 mice (Figure 3B and D). Contrary to Beclin-1 and p62, no change was observed in expressions of LC3-I and LC3-II (Figure 4).

As protein changes of autophagy were observed only at 12 months of age, a transcriptional analysis was performed to complete this monitoring and data showed no significant difference for all markers between APPswePS1dE9 and WT at 12 months of age (Table 1).

**Table 1:** **Monitoring of the mRNA autophagy marker levels in APPswePS1dE9**

	**Wild-type**		**APPswePS1dE9**	
	**Cortex**	**Hippocampus**	**Cortex**	**Hippocampus**
Beclin-1	1.00 ± 0.26	1.00 ± 0.18	1.22 ± 0.21	0.91 ± 0.05
p62	1.00 ± 0.12	1.00 ± 0.08	0.93 ± 0.12	1.10 ± 0.06
LC3	1.00 ± 0.07	1.00 ± 0.08	0.79 ± 0.05	0.82 ± 0.09

Total RNA was isolated from cortex and hippocampus of 12-month old APPswePS1dE9 and WT mice and TaqMan real-time PCR was performed in an ABI 7500 real-time PCR system as described in Materials and methods. Results were normalized relatively to mean of WT mice (n = 12).

**Figure 5:**
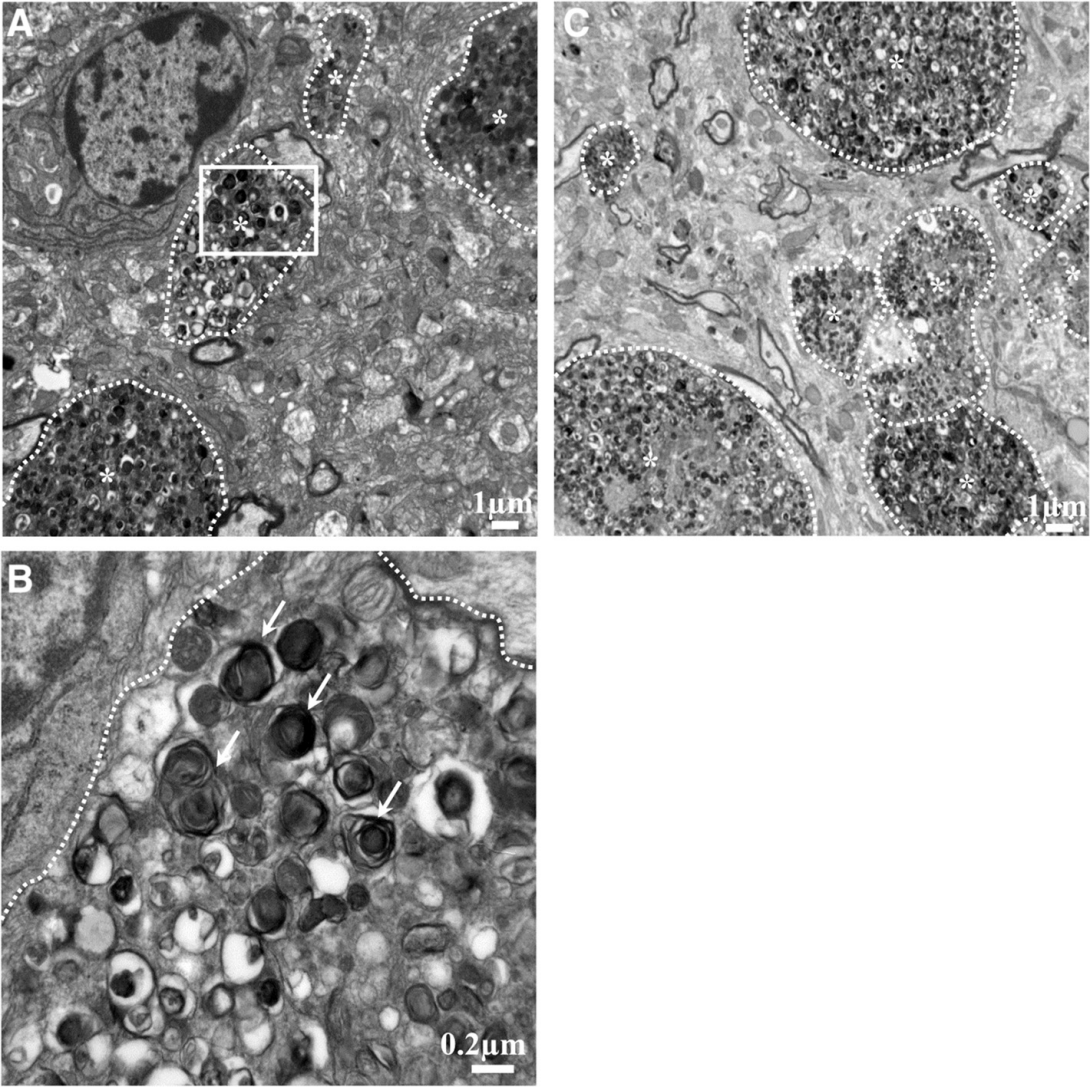
**Ultrastructure of cortex and hippocampus in 12-month old APPswePS1dE9 mice.** Transmission electron microscopy (TEM) cortical **(A)** and hippocampal **(C)** representative images (n = 3 mice) showed dystrophic neurites (marked by *) with a large accumulation of autophagic vesicles (AVs). **(B)** represents a magnified region of interest (defined by a white square in image (a)) within many AVs with a dense compacted amorphous or multilamellar content as indicated by white arrows.

In addition, TEM revealed the presence of dystrophic neurites with a large accumulation of autophagic vesicles (AVs) with a dense compacted amorphous or multilamellar content both in cortex and hippocampus in APPswePS1dE9 mice at 12 months of age (Figure 5). No AVs and no dystrophic neurites were depicted at three and six months of age in these transgenic mice (data not shown).

### Correlation between Beclin-1 and IL-1β levels in cortex and hippocampus of APPswePS1dE9 mice

**Figure 6:**
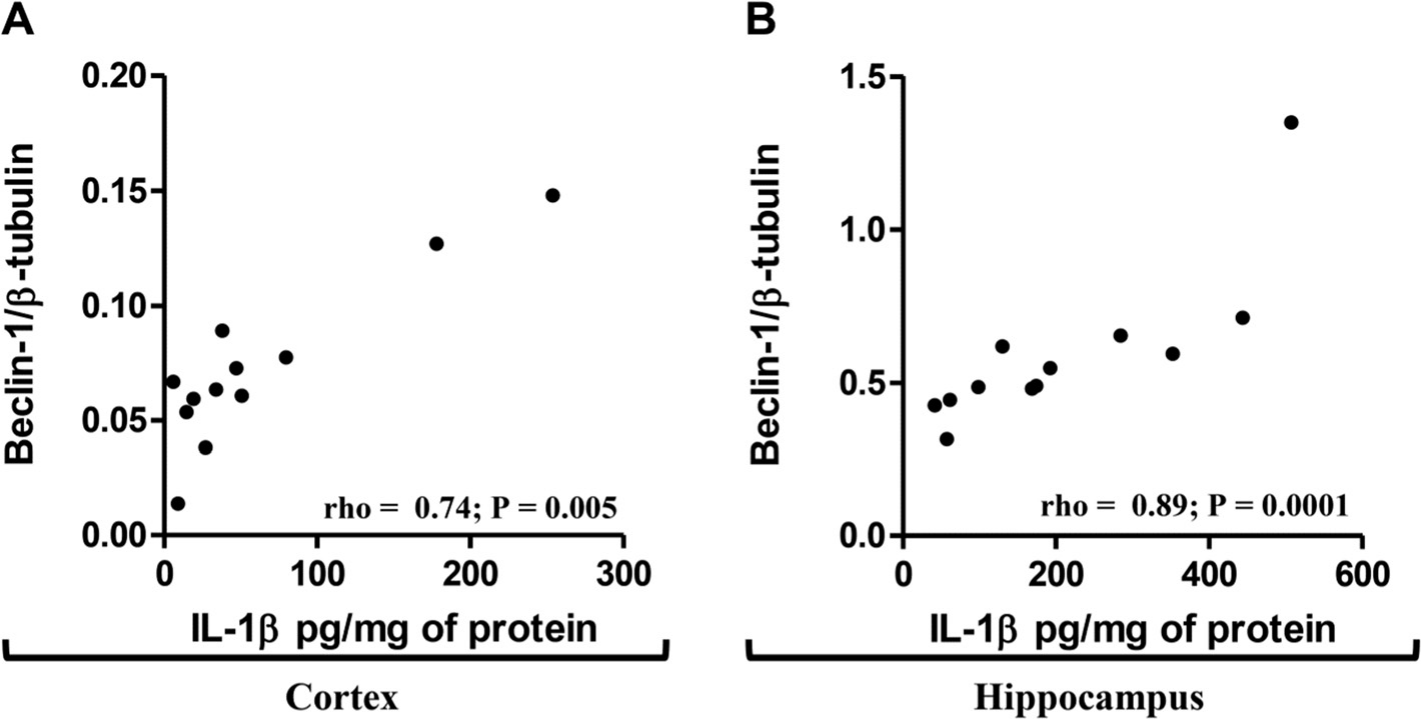
**Correlation between Beclin-1 and IL-1β levels in cortex and hippocampus of 12-month old APPswePS1dE9 mice.** Spearman correlations were performed between levels of IL-1β and Beclin-1 in the cortex **(A)** and hippocampus **(B)** of 12-month old APPswePS1dE9 mice (n = 12). rho and *P-*values were indicated on graphs. The level of significance was *P* < 0.05.

In the 12-month old APPswePS1dE9 mouse group, inflammation and autophagy were significantly correlated. Indeed, Beclin-1 and IL-1β levels in cortex and in hippocampus were positively correlated (Figure 6). In addition, Beclin-1 levels were positively correlated with those of TNF-α in cortex (rho = 0.60; *P* = 0.007). These correlations mean that in the APPswePS1dE9 group, the higher the IL-1β and TNF-α levels, the higher the Beclin-1 expression.

Relationships between mTOR signaling pathway and cytokine production were observed in 12-month old APPswePS1dE9 mice. In hippocampus, TNF-α levels were inversely correlated with the mTOR activation (rho = − 0.46; *P* = 0.04). In cortex, IL-1β and TNF-α levels were inversely correlated with the p70S6K activation (rho = − 0.48; *P* = 0.04 and rho = − 0.49; *P* = 0.03, respectively). These correlations indicate that the higher the IL-1β and TNF-α levels, the lower the mTOR signaling pathway activation. Another interesting result is that cortical levels of Beclin-1 expression were positively correlated with those of p62 (rho = 0.75, *P* = 0.0002). This last correlation would strengthen the results demonstrating a blocking of autophagic flux in 12- month old APPswePS1dE9 mice.

## Discussion

There is mounting evidence that the control of autophagy is impaired in AD as well as in many other neurodegenerative diseases with protein aggregation [43,44]. Furthermore, many studies have revealed that the stimulation of autophagy can reduce Aβ accumulation and alleviate memory deficits in transgenic AD mice [45-48]. As with many other neurodegenerative diseases, AD is also marked by a large inflammatory response and recent data are consistent with the hypothesis that Aβ-induced activation of the NLRP3 inflammasome enhances AD progression by mediating a harmful chronic inflammatory tissue response [11-13].

The crosstalk between autophagy and inflammation is mainly described at peripheral level in particular in inflammatory bowel diseases [49], type 2 diabetes [50], cardiac disorders [51] and cystic fibrosis [52]. Recently, we showed, in primary cultures of neurons, astrocytes and microglia, that IL-1β induced autophagy with accumulation of many acidic vesicles loaded with autophagic markers (p62 and LC3) in microglia, whereas Aβ42 prevented the effects of exogenous IL-1β in the production of inflammatory factors and in autophagy impairment [53]. In this present study, we wanted to explore correlations between autophagy and inflammation *in vivo* by using APPswePS1dE9 mice which displayed amyloid plaques and an inflammatory response at an early stage of the life and progressively increase with age [37,38]. However, their longitudinal status of autophagy has never been studied.

In three, six- and twelve-month old mice, results showed that mice displayed a large increase in IL-1β and TNF-α, a significant decrease in mTOR (a negative regulator of autophagy) and Beclin-1 associated with an accumulation of autophagic vacuoles (AVs) with a dense compacted amorphous or multilamellar content at twelve months of age in hippocampus and cortex. Interestingly, correlation analyses showed that Beclin-1 was positively correlated with IL-1β and TNF-α and these cytokine levels were inversely correlated with the levels of mTOR activation at 12 months of age.

The monitoring of inflammation in APPswePS1dE9 versus WT mice at 3, 6 and 12 months of age showed that APPswePS1dE9 mice displayed cytokine production, in particular IL-1β in cortex at 12 months of age and hippocampus from 6 months, but TNF-α levels increased only in hippocampus at 12 months and were less than 10 pg/mg protein as previously described [37,38]. In line with our work, Jin *et al*. did not find any modification of the levels of IL-6. Similar changes in cytokine expression have been shown in other transgenic mice and reflected those observed in AD patients [54].

Concerning autophagy, it is well-known that the mTOR activation is a primordial inhibitory signal [55-59]. In these APPswePS1dE9 mice, a large decrease of the activation of mTOR and its downstream substrate p70S6K was observed according to previous findings in other transgenic mouse AD models [60-63]. However, in some transgenic mouse models of AD, the mTOR activation was not modified while the p70S6K activation decreased [64]. In two independent mouse models of AD, rapamycin, through inhibition of mTOR signaling, rescued cognitive deficits by suppressing extracellular Aβ deposition and intracellular tau accumulation [45,63]. Interestingly, this treatment induced autophagy marked by increased expression of Atg 7, Atg5-Atg12, decrease in p62, no modification of LC3 expression and controversial results surrounding LC3-II expression in PDAPP and 3xTg-AD mice [45,46]. In our experimental conditions, APPswePS1dE9 mice not treated with rapamycin displayed a large inactivation of mTOR signaling pathway from 6 months of age in hippocampus, a significant decrease of Beclin-1 both in cortex and hippocampus and p62 in cortex at 12 months of age, but no modification of LC3-I and LC3-II expression. These decreases of autophagic markers were significantly different from mice at three and six months of age and were associated with extensive accumulation of autophagic vesicles either with a dense compacted amorphous or multilamellar content within dystrophic neurites as previously described in other AD transgenic mice [35,46,65,66] and in brains of AD patients [29].

Recent studies have demonstrated that the *p62* gene expression and cytoplasmic p62 protein levels are significantly reduced in the frontal cortex of AD patients compared to that of control subjects [32,67]. Here, no modification of transcriptional expression was observed while a large decrease of its protein expression was quantified only in cortex of APPswePS1dE9 mice as in cortex of AD patients. This decrease could be explained by its sequestration into amyloid aggregates reducing the accessibility of p62 protein for the regulation of signaling, trafficking and autophagic clearance of ubiquitinated proteins [68]. Moreover, studies in p62 knockout mice have clearly demonstrated that the lack of p62 protein leads to the neuropathological lesions including the accumulation of hyperphosphorylated tau and neurofibrillary tangles, synaptic deficiencies with loss of working memory and neuronal apoptosis [69].

In addition, it was shown that an inhibition of p62 resulted in the formation of mis-regulated autophagosomes with multilayer membranes and an autophagic cell death in carcinoma cells [27]. The expression of p62 is also determined by availability of lysosomal-derived amino acids used for *de novo* synthesis of p62 [70]. However in AD, it is well-known that lysosomal acidification is disrupted [71,72].

The reduced Beclin-1 protein levels may be related to caspase-cleavage as shown in frontal cortex tissue from moderate to severe AD cases [66,73-75]. Some miRNA, miR-30a and miR-376b target Beclin mRNA [76,77] and miR-30a levels have been shown to increase in cerebrospinal fluid of AD patients [70]. Another explanation for the reduced expression of Beclin-1 could be a non-functional sequestration of Beclin-1 as it is a molecular platform assembling an interactome, with stimulating and suppressive components, which regulate the initiation of the autophagosome formation [75]. In AD transgenic mice, Beclin-1 reduced neuronal autophagy, disrupted lysosomes, promoted intracellular and extracellular Aβ accumulation, and resulted in neurodegeneration [35]. On the contrary, increased expression of it reversed these impairments in mice [78,79]. Recent studies position Beclin-1 as a link between autophagy, retromer trafficking, and receptor-mediated phagocytosis [80]. Indeed, Beclin-1, together with its phosphatidylinositol 3-kinase (PI3K) binding partner, Vps34, played a role in receptor-mediated phagocytosis by regulating the retromer complex in microglia [80].

Interestingly, results showed positive correlation between Beclin-1 and IL-1β in cortex and hippocampus and with TNF-α in cortex, suggesting relationships between inflammatory response and autophagy impairment in 12-month old APPswePS1dE9 mice. It is known that many inflammasomal receptors, in particular NLRP4, could interact with Beclin-1 and inhibit autophagocytosis [81]. Furthermore, the levels of these cytokines were inversely correlated with the activation of mTOR signaling pathway. It is known that the mTOR signaling pathway can inhibit the inflammatory response in microglia and monocytes by reducing NF-κB activation and enhancing STAT3 activity and anti-inflammatory IL-10 production [82,83], whereas inhibition of mTOR with or without rapamycin has reciprocal effects [84-86]. Therefore, in the APPswePS1dE9 mouse model, the inhibition of the mTOR signaling pathway could participate in the inflammatory response which could impair autophagy. However, mTORC1 colocalizes with the Transcription Factor EB (TFEB), a master regulator of lysosomal biogenesis on the lysosomal membrane, leading to inhibition of TFEB activity by mTORC1-induced phosphorylation. Conversely, pharmacological inhibition of mTORC1, as well as starvation and lysosomal disruption, activates TFEB by promoting its nuclear translocation for lysosomal biogenesis and autophagy [87-90]. However, in the APPswePS1dE9 mouse model there was an accumulation of AVs that may have been due to a dysfunction of lysosomal activity. It is known that mutation of PS1 leads to the accumulation of immature unglycosylated v-ATPase which is needed in the acidification of autolysosomes and/or lysosomes with abnormal accumulation of late-stage autophagosomes with undigested contents just like the ultrastructures present in AD neurons [71,72,91]. Recently, inhibition of GSK-3β or cystatin B, an endogenous cathepsin inhibitor, could restore lysosomal acidification that in turn enables clearance of Aβ burdens and reactivation of mTOR. These changes facilitate amelioration in cognitive function in 5×FAD and TgCRND8 mice [48,60].

For LC3, no variation is observed in APPswePS1dE9 mice regardless of age compared to WT mice. Other authors show no modification of LC3-I [45,46]. For LC3-II isoform, the results in the literature are contradictory despite the absence of variation of LC3-I. Indeed, some have observed an increase in the expression of LC3-II at a very late age of 18 months in 3xTg-AD treated with rapamycin, which is known to inhibit mTOR and thus induce autophagy [46]. Other APP/PS1 mice (PS1M146L/APP751SL) aged 18 months also showed an accumulation of LC3-II in microsomal fractions and very little in the synaptosomes [92]. Other authors showed a decrease of LC3-II in PDAPP mice at the age of eight to nine months although treated for thirteen weeks with rapamycin since the age of four months [45].

Therefore, the expression rate of LC3-II varies depending on the age of the mice and its subcellular localization. Furthermore, we cannot exclude a variation depending on the transgenic AD model.

## Conclusion

Taken together, these results highlighted the negative impact of IL-1β and TNF-α in the activation of the mTOR signaling pathway (negative correlation) and in the induction of autophagy (positive correlation with Beclin-1) which remained locked and led to the accumulation of AVs in APPswePS1dE9 at 12 months of age. This first demonstration of the relationships between inflammation and autophagy *in vivo* should into account in new therapeutic strategies to prevent inflammation and/or stimulate autophagy in advanced neurodegenerative process such as AD. Furthermore, development of similar but inactive molecules to Beclin-1 could limit its non-functional sequestration and allow the maintenance of a sufficient level of free active Beclin-1 to initiate autophagic flux.

## Abbreviations

AD: Alzheimer’s disease
Aβ: β-amyloid peptide
APP: amyloid precursor protein
AVs: autophagic vesicles
bp: base pair
BSA: bovine serum albumin
CNS: central nervous system
DTT: dithiothreitol
ELISA: enzyme-linked immunosorbent assay
FAD: familial Alzheimer’s disease
FAM: 6-carboxy-fluorescein
HRP: horseradish peroxidase
Ig: immunoglobulin
IL: interleukin
ip: intraperitoneally
LC3: microtubule-associated protein 1/light chain 3
LDS: lithium dodecyl-sulfate
LPS: lipopolysaccharide
mTOR: mammalian Target of Rapamycin
NaF: sodium fluoride
NF-κB: nuclear factor-kappa B
NLR: nucleotide oligomerization domain receptors
OD: optical density
PCR: polymerase chain reaction
PBS: phosphate- buffered saline
PFA: paraformaldehyde
PI3K: phosphatidylinositol 3-kinase
p62/SQMT1: sequestosome 1
PMSF: phenylmethylsulfonyl fluoride
PrP: prion protein
PSEN1 or PS1: presenilin 1
RT: room temperature
SDS: sodium dodecyl-sulfate
TAMRA: 6-carboxytetramethylrhodamine
TBST: Tris-buffered saline/Tween
TEM: transmission electron microscopy
TFEB: Transcription Factor EB
TMB: tetramethylbenzidine
TNF: tumor necrosis factor
WT: wild-type
